# Connecticut Valley and Massachusetts Dental Societies

**Published:** 1888-10

**Authors:** Ira G. Baumgardner


### AFTERNOON SESSION, JULY 10TH, 1888.

UNION MEETING OF THE CONNECTICUT VALLEY AND MASSACHUSETTS DENTAL SOCIETIES, JULY, 1888. REPORTED FOR THE "INDEPENDENT PRACTITIONER" BY IRA G. BAUMGARDNER, D.D.S.

DISCUSSION ON PAPER READ BY DR. S. S. STOWELL, ON "THE USES OF COPPER AMALGAM."

*Dr. E. A. Bogue*, New York City—I do not know that, unprepared, I shall be able to say anything on the subject now before the meeting. I did think while the essayist was reading the paper, that what seemed to me a slight exaggeration in his view of mate. rial might, perhaps, be corrected. It is true that copper amalgam has been used for many years with great suceess and benefit. It has also been shown that certain specimens of this material have proven themselves destructive. What specimens are destructive, and what are not, I am unable to say. My assistant, Dr. Rodgers, showed me, more than a dozen years ago, fillings in his mouth that his father put in twenty-two years previous, using copper amalgam. They were on the grinding surfaces and were good. Within the last five years I have seen a good many copper amalgam fillings that bore an appearance, as far as distinctibility is concerned, like oxyphosphate; they were cupped on the surface and granular. It is true that copper amalgam will not shrink. It is also true that palladium will not shrink. It is not, I think, quite true, as I have already said, that copper is indestructible. It is an oxidizable metal, while palladium—one of the noble metals—is not oxidizable. It is true that copper may be manipulated so as to set rapidly. Palladium can scarcely be manipulated in any other way but rapidly, so that contour fillings can be made with it. When finished, both are intensely black.

There was one remark Dr. Stowell made I should like to take exception to, that he could face his copper amalgam with what he has called "high grade" amalgam, or gold filling. As I understand it, the copper amalgam has no affinity for another amalgam, and that if union does take place it is purely a mechanical one. If it is anything else I should like to learn it. I have never had any amalgam adhere.

Another thing he said was that the patient should be cautioned not to bite upon copper amalgam fillings for several hours. I think we should be pretty careful not to put any amalgam filling into teeth where the biting surfaces are concerned. It seems to me it is not a substance we can safely put on the biting surface, or where the opposing teeth come together. The amalgam is intensely hard and teeth elastic, and I have seen many teeth split in that process Dr. Stowell alluded to. Perhaps he endeavored to make the same point I do. Finally, Dr. Stowell refers to the antiseptic properties of copper. Inasmuch as I must regard all the bacterial inhabitants of the mouth as scavengers, I earnestly hope that copper will never be used in the mouths of our patients, relying absolutely upon the independent cleanliness of the copper.

*Dr. S. J. Andres*, Montreal, Canada—I never had any experience in using copper amalgam except when I was a young man studying the profession of dentistry, and beginning practice, a great many years ago. The only thing we used was the old "Spanish Quarter," making a filling that stood then, and will now, better than anything else. I have seen filling that had been in the mouth thirty-five years, and standing firm. I think that in the alloy of the Spanish quarter, silver and copper were the only two metals used.

*Dr. G. H. Weagant*, Cornwall, Conn.—I had the honor, last year, at Montreal, of reading a paper on the same subject, and I think covered all the points made to-day; and I believe I agree with Dr. Stowell in everything he said upon the subject, with the exception that I agree with Dr. Bogue in reference to making ordinary amalgams adhere to copper amalgam. The only way is to introduce one over the other. They will not adhere to copper amalgam.

In reference to the point Dr. Bogue spoke about, the copper looking like oxyphosphate filling, I think it was entirely due to impurities in the amalgam. That has been my experience. If the copper amalgam is perfectly clean, I do not think any such condition as Dr. Bogue mentions need occur.

*Dr. B. A. R. Ottolingui*, New York City—In regard to copper amalgam, it seems singular that so much has been said in favor of it and so little against it. The result is younger men in the profession are likely to run into it to get out of their mistakes. It may be that copper amalgam is a fine germicide to stop decay, but it can only stop where it touches; and if a person is uncleanly, their teeth may decay where the amalgam does not touch, and they may fail when they reach that cavity, and the copper amalgam will not stop it from going under it. I allude to places where you put a copper amalgam in a posterior cavity of any tooth—lower molar, say — and then if, from uncleanly habits of the patient, a cavity forms decays on the linguial aspect, and burrows along the margin of the gum, and then under that filling, in the course of time the patient will come back with the filling loose. It is then necessary to take that filling out and refill that tooth properly, which is difficult for us to do, for the filling is so hard. This remark is not original with me; it was made by some other gentleman, but I think it is a well taken point to warn us that when we do anything of this kind, to look out for the shoals.

I have not had much experience in using copper amalgam myself. I have only used Dr. Russell's, of Brooklyn. It has not enough mercury to make a stopping. I understand Dr. Russell puts it under a hydraulic press, gets out the mercury, and then heats it up, and then renews the pressing of it three times. In this way the quantity of mercury is reduced to a minimum, and you have to add mercury to it to put it in.

In regard to facing the filling, you cannot work amalgam upon it well or use foil upon it, but you can burnish Watt's crystal gold into it. Whether that gives a good color I have not had time to find out.

*Dr. J. H. Kidder*, Lawrence, Mass.—A brother of mine, twenty years older than myself, one of the older practitioners of New Hampshire—even when amalgam was under a cloud—invariably stuck to his own experience, and filled teeth with it right along. I have now in my mouth fillings that he put in—made from silver dollars—that have been in there about fifty years. Some other portions of the teeth have been filled, and others of my teeth were filled with soft gold, which were not removed until several years ago. I then had them filled with oxyphosphate, on account of their getting old and frail. As far as amalgam is concerned, I think it makes a good filling.

*Dr. H. W. Clapp*, Westfield, Mass.—I have very little experience with copper amalgam. I have used it a little for perhaps eighteen months, and so far as my experience goes I am very much pleased with it.

*Dr. W. X. Sudduth*, Philadelphia—This is a practical discussion which is almost entirely out of my sphere, yet the question was raised regarding the germicidal properties of copper amalgam. I have made a few tests in that regard, and my judgment is that its germicidal properties are very low indeed. Certain conditions may be brought about by which chemical changes will take place, and in that case it may have slight power as a germicide; but as a dependent I think its value is more due to non-shrinkage and adaptability, by which it forms a perfect stopping for teeth, thus preventing entrance of the micro-organisms into the cavity.

*Dr. G. A. Maxfield*, Holyoke, Mass.—I am a little disappointed in the way the discussion has progressed, as I was in hopes we would get a little more of the practical experience that some of the gentlemen have had. In my practice I want to use what is the best material, whether copper, gold or common alloy. I have not used copper amalgam very long. Dr. Weagant gave us an excellent paper at Montreal last summer, and since then I have experimented somewhat with it. I took his formula and made some for myself. I found I had an amalgam which was much the same as some that has since been placed on the market, which —after heating and crushing in a mortar—forms a dry powder, and we have to add more mercury to it. If, however, you heat it up three or four times, and work it thoroughly, it makes a very plastic filling, showing that there is plenty of mercury in the filling. The trouble was that we broke up the crystals by grinding in the mortar. If we can keep that crystallization, and not break it up, we get an amalgam that will set very readily, perhaps in ten minutes.

Now, as regards using it in combination with other alloys, I have done this : Use a matrix and hammer it in as well as possible with bibulous paper, and then I take my other alloy and put it in the same way. It may be a mechanical union, but the fillings remained solid. I cannot say how long they will last, for it is only nine months since I began using the combination; but I saw some last week that had been placed in nine months ago that were as solid as when put in the mouth. The alloy on the surface has not changed color. There is a black shade where the two unite, but does not go up into the other amalgam. In regard to making the amalgam quick setting, I find that if you let the mercury just bubble, then you have a very dry amalgam.

*Dr. S. S. Stowell*, Pittsfield, Mass.—I wish to say a word in defence of my paper. In regard to the topping off of these amalgams with other alloys, my experience has been the same as Dr. Maxfield's. Whether there is a perfect union of metal I am not prepared to say. I only know I have made the combination filling without any failure or apparent separation of the top from the other. I simply know I have no failures of that kind that have come under my observation; and in regard to the filling becoming granular and cup-shaped, as Dr. Bogue has mentioned, I must claim that, when that result followed its use, it is due to faulty manipulation of amalgams. This amalgam may be heated and rubbed in a mortar, and become as dry as sand; and you begin to think that there is not sufficient mercury. The surplus of mercury is there; and, if you add any, there will be too much. If you put it back in the spoon and heat the amalgam again, you can produce a soft and plastic amalgam.

In regard to the introduction of this copper amalgam—the general use of it in this country—one dealer told me yesterday that, four years ago, he imported five ounces of the English copper amalgam. His men on the road took it around and showed it to dentists, and they did not want it. He now sells an average of two hundred ounces of copper amalgam annually. If used so extensively in this day of research and advancement, there must be something in it.

*Dr. E. A. Bogue*—Dr. Stowell states that the profession is now using two hundred ounces of copper amalgam where formerly four or five were sold. I think that reflects somewhat upon us as a craft. We are a little too apt to think of the last new thing, and run after it possibly too fast. Now, while I did not reach my majority until a few years ago—three or four—I am Dr. Stowell's grandfather so far as copper amalgam is concerned. When Dr. Stowell speaks of a combination filling, I am unable to comprehend what he means. If he means a cavity shaped like a well, not a well-shaped cavity, I can better understand him. Filling a posterior cavity open on two sides is a different thing. In the former case I can readily understand how he would get a combination filling that would show no disintegration between the two different metals; but in the latter case, a posterior proximal cavity, I cannot see how a filling would adhere or cohere with that amalgam, and would be interested in knowing how to do that thing.

Dr. Stowell speaks of faulty manipulation of copper amalgam being the cause of cupping. He will pardon me, I hope, for saying that fourteen years ought to give one some experience in manipulation; but I have had these fillings go back on me, and I wish to give a word of warning. I do recognize the great value of it in many places where other materials would be inadmissible. In all places hidden from view, where thermal changes would not be felt, there use copper, unless in proximal cases, where you cannot get it out again. There I should want to use something else.

I prepare copper amalgam exactly as Dr. Maxfield has described; and if there is a reasonable amount of mercury in it, it becomes, in my mortar, soft, although it may be so porous and granular that it seems impossible to get that copper into a mass. I then place in the bottom of the cavity Fletcher's copal varnish, so as to stick it in—in the tooth, of course. Then I put in my copper amalgam quite soft. The balance is squeezed out as hard as I can get it, and if that does not satisfy me, I heat crystal gold in my alcohol lamp, and put on the surface until I have sponged out with the gold all the mercury I can get out. I then burnish the amalgam, and then have it almost as hard as I want.

*Dr. S. J. Andres*—I have seen somewhere the assertion that the use of gold in any shape to absorb mercury from an amalgam filling would injure it. I have used Watt's crystal gold for nearly two years to take up the surplus mercury. If it injures the filling I have been using it wrongly, and I should like to be set right.

*Dr. Bogue*—It will cause the filling to shrink.

*Dr. J. G. Werner*, Boston, Mass.—Some one has expressed the idea that he is not a "copperhead" to new things. It is the superficial theorist who takes the profession so readily. Because it is 200 oz. it should have no weight with the young men. I have not used the article, and am pretty sure I shall not until I hear more of it, and I think the young men should be cautioned.

*Dr. S. S. Stowell.*—I have always selected my cases. I do not use it when the teeth are hard and strong. I use it to accomplish a thing I could not accomplish in any other way—in teeth that are chalky and soft, and seem to defy our best efforts by other means.

*Dr. G. A. Maxfield*—In regard to the 200 oz. of copper amalgam, I think as a rule our profession is very conservative. There has been a great prejudice against copper amalgam because it is black. I was of the same opinion, and would not use it until I heard Dr. Weagant's paper last year. I think one thing that has brought about such a large sale of copper amalgam was the statement of Dr. Miller. He made a series of very careful tests, and demonstrated that copper amalgam was the only filling around which the gums will not live. I think the profession will take that as reliable. There are very few of us who can make these tests. We have got to go by teachers. If they are reliable we can then adopt the different materials. We do not know how some of our patients clean their teeth, and we must treat their teeth in such a way that the germs will not start decay again.

*Dr. G. F. Waters*, Boston, Mass.—I have noticed that all of those who have spoken of this copper amalgam state they put it into the hand and work it. One statement made was that if there were impurities in the amalgam, then it would not stand well. What is the result of working amalgam in the hand? Your hand is full of pores, more upon the palmar surface than on the back side, and which are affected by alterations of pressure. The glands are thus brought into a state of active secretion, and the effete matter of the body is thrown to the surface. You are mixing with that amalgam carbonic acid, and perhaps carbonic oxide and perhaps sodium, and possibly chloride of sodium, and what is the result? It certainly is not a pure mass you are putting into the tooth. I never mix amalgam in my hand, because it is not possible to obtain a pure mass in that way.

*Dr. D. D. Peabody*, Stoneham, Mass.—I will say, in answer to Dr. Waters, that if he will cover the hand with rubber dam and cover the finger with kid, the difficulty mentioned can be easily overcome.

[To be continued.]

